# Nano-Antibacterials Using Medicinal Plant Components: An Overview

**DOI:** 10.3389/fmicb.2021.768739

**Published:** 2022-02-22

**Authors:** Sourav Ghosh, Susmita Nandi, Tarakdas Basu

**Affiliations:** Department of Biochemistry and Biophysics, University of Kalyani, Kalyani, India

**Keywords:** medicinal plants, major components, nanonization, antibacterials, mechanism of action

## Abstract

Gradual emergence of new bacterial strains, resistant to one or more antibiotics, necessitates development of new antibacterials to prevent us from newly evolved disease-causing, drug-resistant, pathogenic bacteria. Different inorganic and organic compounds have been synthesized as antibacterials, but with the problem of toxicity. Other alternatives of using green products, i.e., the medicinal plant extracts with biocompatible and potent antibacterial characteristics, also had limitation because of their low aqueous solubility and therefore less bioavailability. Use of nanotechnological strategy appears to be a savior, where phytochemicals are nanonized through encapsulation or entrapment within inorganic or organic hydrophilic capping agents. Nanonization of such products not only makes them water soluble but also helps to attain high surface to volume ratio and therefore high reaction area of the nanonized products with better therapeutic potential, over that of the equivalent amount of raw bulk products. Medicinal plant extracts, whose prime components are flavonoids, alkaloids, terpenoids, polyphenolic compounds, and essential oils, are in one hand nanonized (capped and stabilized) by polymers, lipids, or clay materials for developing nanodrugs; on the other hand, high antioxidant activity of those plant extracts is also used to reduce various metal salts to produce metallic nanoparticles. In this review, five medicinal plants, *viz*., tulsi (*Ocimum sanctum*), turmeric (*Curcuma longa*), aloe vera (*Aloe vera*), oregano (*Oregano vulgare*), and eucalyptus (*Eucalyptus globulus*), with promising antibacterial potential and the nanoformulations associated with the plants’ crude extracts and their respective major components (eugenol, curcumin, anthraquinone, carvacrol, eucalyptus oil) have been discussed with respect to their antibacterial potency.

## Introduction

Bacteria are ubiquitous in nature and play an important role to keep up the homeostasis in the environment in which we live (Doron and Gorbach, 2006). Bacteria have both beneficial and harmful effects in public health. So far as their helpful roles are concerned, they provide vital ecosystem services. Bacterial species like *Bacillus subtilis, Pseudomonas fluorescens*, and others decompose dead organisms to release inorganic elements to maintain the balance in continuity of carbon and nitrogen cycles and thus clean the environment (Sivasakthi et al., 2014). Bacteria are very much useful for civilization in the aspect of multiple purposes such as for production of ethanol, enzymes, antibiotics, and biogas; for fermenting cheese and yogurt; for cleaning of oil spills and toxic wastes; and also in many more fields (Raaijmakers et al., 2002; Ma et al., 2009; Das and Chandran, 2011; Youngsukkasem et al., 2012; Singh and Singh, 2014; Li et al., 2020). The human gut is colonized by 10^14^ microbes, out of which bacteria consist of a major part as commensal ones (Zhang et al., 2015). Gut bacteria provide essential nutrients, synthesize vitamin K, help in digestion of food stuff dietary fibers-polyphenols, and also encourage angiogenesis and enteric nerve functions (Tsuji et al., 2008; Hill and Artis, 2010). Breakdown of gut environmental homeostasis leads to dysbiosis of bacteria, which may cause different types of illness like allergy, inflammatory bowel disease (IBD), obesity, diabetes, and even cancer (Ticlla et al., 2021).

On the other hand, bacterial species like *Mycobacterium tuberculosis*, *Vibrio cholerae*, and *Salmonella typhimurium* having a deleterious role of causing diseases are called pathogenic bacteria. Development of antibacterials is necessary to combat the pathogenicity of such disease-causing bacteria. The disease tuberculosis, caused by *Mycobacterium tuberculosis*, spreads through contaminated air and approximately 27,000 people are affected by tuberculosis with about 4,000 deaths per day from this disease (Yamano, 2019). WHO listed two bacterial pathogens *Pseudomonas aeruginosa* and *Acinetobacter baumannii* as a public health threat for developing nosocomial disorders like pneumonia, sepsis, bacteremia, urinary tract infections, and various complications in lungs (Mwangi et al., 2019). An important life-threatening bacterial disease is cholera, which is caused by *Vibrio cholerae* and is transmitted by contaminated food and water. As per WHO report (February 5, 2021), about 1.3–1.4 million cases of cholera infection occur globally each year and the number of deaths ranges from about 21,000–143,000 from country to country. An important water-borne, *Salmonella typhi*–mediated fatal disease is typhoid fever, by which about 21 million illnesses and 2,16,500 deaths occurred globally in 2000, affecting all age groups (Bhan et al., 2005). A fulminant zoonotic complication “plague” is caused by the bacteria *Yersinia pestis* and *Yersinia pseudotuberculosis*, and in the history of earth, plague had been appeared multiple times as epidemic (Prentice and Rahalison, 2007). Another major health problem in most developing countries is dysentery that is caused by different enteropathogenic bacteria such as *Shigella flexneri* and *Shigella dysenteriae*. Dysentery causes more than 1 million deaths globally each year and children specifically under 5 years are more susceptible for the infection (Mukherjee et al., 2019). Besides these, some clinically relevant infections are meningitidis (a disease of brain characterized by fever, headache, vomiting, inability to lower chin to chest, non-specific maculopapular rash, joint pain, sign of vasculitis, and also appearance of conjunctivitis, panophthalmitis, and pneumonia caused by *Neisseria meningitides*; Stephens et al., 2007) and sexually transmitted diseases like syphilis and gonorrhea caused by *Treponema pallidum* and *Neisseria gonorrhoeae*, respectively (Workowski et al., 2015). Thus, bacterial infections have a great impact on public health, which is a fundamentally important issue for maintaining the stability and wellbeing of a nation or region.

An important phenomenon of bacterial infection is biofilm formation. Bacteria alternate between two forms—free-living planktonic and surface-attached biofilm—depending on the environmental conditions. Biofilm is a bacterial assemblage enclosed in self-produced extracellular polymeric substance (EPS), which is made up of extracellular DNA, polysaccharides, and proteins. EPS prevents the direct exposure of biofilm-residing cells to the different types of antibacterials. Biofilm causes more than 80% of chronic infections like pneumonia in cystic fibrosis patients, chronic wounds, chronic otitis media, and organ implant and catheter-associated infections (Bjarnsholt, 2013). Such infections affect millions of people globally with consequent death of millions of people each year (Bjarnsholt, 2013).

**FIGURE 1:**
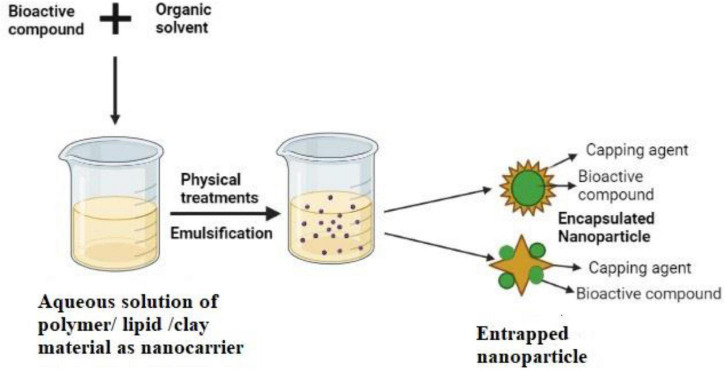
Schematic representation of active compound loaded nanoparticle.

To prevent bacterial infections, there are various approaches. The conventional antibacterial approach is the use of antibiotics, but most of the clinically important bacterial infections are gradually going to be antibiotic resistant. Biofilm infections are the hallmark of antibiotic resistance (Levy and Marshall, 2004). Antibiotics work in a specific way so that a particular cellular process (synthesis of cell wall, protein, DNA, and RNA) is disturbed and therefore bacteria become easily antibiotic resistant by acquiring mobile genetic elements such as bacteriophages, plasmids, naked DNA, transposons, or through chromosomal mutation also (Milkman, 1990; Schneiders et al., 2003). The problem of antibiotic resistance generation in bacteria led to the exploration of the synthesis of different inorganic (particularly metals and metal oxides like Au, Ag, Cu, Zn, CuO, ZnO, MgO, and TiO_2_; Saidin et al., 2021) and organic (chlorohexidine, triclosan, polyaniline, polyethylenimine) molecules as antibacterials (Saidin et al., 2021). Such molecules were found to combat infection of a broad spectrum of bacteria like *Escherichia coli*, *Staphylococcus aureus*, *Klebsiella pneumoniae*, *Salmonella typhimurium*, and many others. They attack the bacteria through various routes, *viz*., membrane disruption, DNA damage, protein synthesis inhibition, and free radical generation (Soenen et al., 2011); however, the major problem of their use is cytotoxicity, which is generated by excessive ROS (reactive oxygen species) production, changes in cell morphology with cytoskeleton defects, introduction of genotoxicity, and so on (Soenen et al., 2011). To annihilate the problem of cytotoxicity, green products, especially the medicinal plant components, represent a better biocompatible platform with significant antibacterial potential (Sdrolia and Zarotiadis, 2018). However, most of such plants’ ingredients are poorly soluble in aqueous media, i.e., poorly bioavailable and are not highly stable too. To make them bioavailable and stable, the use of nanotechnological strategy is increasing, where hydrophobic active components of plants are nanonized through encapsulation or entrapment within inorganic or organic nanocarrier molecules, as shown in the representative Figure 1. Such nanoformulations of medicinal plant products are found to have enormous therapeutic potential against bacterial infection. Nanomedicines developed by this strategy are found to have the property of sustained release of active drug component from nanocarrier molecule and thereby retaining the drug efficacy for a longer period (Sarmukaddam et al., 2010). In addition, medicinal plant extracts are also used as nanocarrier/nanostabilizer to reduce metal salts/oxides of Ag, Au, Cu, and Zn to metallic nanoparticles (NPs) by their high antioxidant activity, producing green synthesized metallic NPs as potential antibacterials (Dubey et al., 2010; Zamare et al., 2016; Mali et al., 2020; Jayachandran et al., 2021). In this article, antibacterial action of five important medicinal plant extracts, *viz*. tulsi (*Ocimum sanctum*), turmeric (*Curcuma longa*), aloe vera (*Aloe vera*), oregano (*Oregano vulgare*), and eucalyptus (*Eucalyptus globulus*), and their respective major components eugenol, curcumin, anthraquinone, carvacrol, and eucalyptus oil in nano-forms has been reviewed.

## Tulsi (*Ocimum sanctum*)

This flowering plant of the mint family (Lamiaceae) is very much common in the Indian subcontinent and grows throughout Southeast Asia. The literal meaning of the term “Tulsi” means incomparable one and this plant is also considered as “queen of herb” because of its diverse healing and health-improving properties to the human body. This medicinal plant is widely used centuries after centuries in traditional medicinal practice. Ancient Indian medical practitioners and scientists described “Tulsi” as an adaptogen, which balances different processes of the body, helps to adapt to the stresses, and boosts energy. Tulsi is regarded as “elixir of life” because of its excellent and versatile therapeutic potentials. Different parts of the plant (leaves, stem, roots, seeds, flowers) are used in traditional medicine sector, but most of the medicinal formulations are based on leaf extract because of its highest adaptogenic and antioxidant activities. Tulsi extract has been recommended to treat malaria, diarrhea, dysentery, skin disease, eye disease, and so on (Pattanayak et al., 2010). The extract has also been found to possess anticancer, hepatoprotective, antiemetic, antispasmodic, analgesic, antidiabetic, and anti-arthritis actions (Prakash and Gupta, 2005). It also reduces the risk of heart attack and lowers the cholesterol level (Bhargava and Singh, 1981; Gupta et al., 2002). The herbal formulation of tulsi is used for asthma, short breath, and also in respiratory ailments like bronchitis and tuberculosis (Das and Vasudevan, 2006).

So far as our knowledge goes regarding nanoformulations using medicinal plant extracts, there is hopefully no report of nanonization of whole extracts of any plant part through encapsulation or entrapment within any nanocarrier molecule, but there are reports about the use of plant extract as stabilizing agent to produce metallic and metal oxide NPs. Tulsi extract has been used to produce and stabilize silver nanoparticles (AgNPs) from the precursor silver nitrate molecules. During such green synthesis (phytoreduction) of silver NP, tulsi leaf extract converts silver ion into elemental silver by reduction of silver nitrate and produces monodispersed, spherical AgNPs of size about 20 nm. Such AgNP preparation is found to have more antibacterial activity on *Escherichia coli* and *Staphylococcus aureus*, compared with the individual precursors silver nitrate and leaf extract. This becomes evident from the size of the zones of inhibition measured to be about 10.5, 0, and 8 mm, respectively, for equivalent concentrations of AgNPs, silver nitrate, and leaf extract (Ramteke et al., 2013). This result implies that both the nanoformulation and the leaf extract have antibacterial activity and the activity of the nanoformulation is about 25% more than that of the leaf extract, whereas silver nitrate itself has no antibacterial activity. Paper towels coated with such tulsi extract–stabilized AgNPs have antibacterial activity against *Escherichia coli*, *Staphylococcus aureus*, and *Klebsiella pneumoniae* (Jacob et al., 2019). Such AgNP-coated cotton and leather surfaces inhibit growth of *Bacillus linens*, *Pseudomonas acnes*, *Bacillus cereus*, and *Staphylococcus epidermidis* (Jacob et al., 2019). AgNPs continuously release Ag^+^ ions, which have strong affinity to cell wall and sulfur proteins. The adhered ions enhance permeability of cytoplasmic membrane and thus lead to bacterial cell wall disruption (Khorrami et al., 2018). After having entered into the cells, silver ion deactivates respiratory enzymes and produces ROS by inhibition of ATP generation (Ramkumar et al., 2017). ROS causes membrane disruption leading to problems in DNA replication and cell division (Yin et al., 2020), as bacterial DNA is believed to remain attached with cell membrane for a considerable time of their life cycle. Silver ion is also reported to inhibit protein synthesis by disintegrating ribosomes (Durán et al., 2016).

**FIGURE 2:**
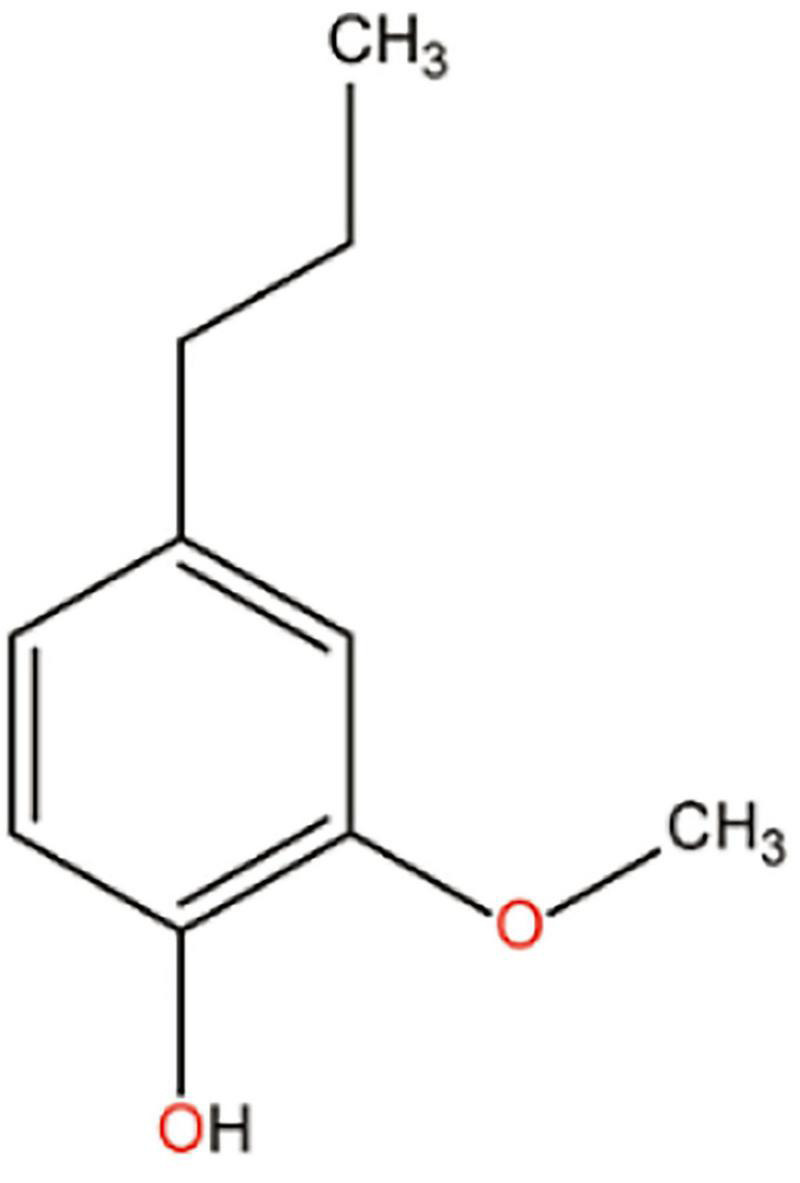
Chemical structure of eugenol.

The phytochemical composition differs in various parts of the plant. The most commonly used leaf extract as medicines contains several volatile oils such as eugenol, euginal, urosolic acid, carvacrol, limatrol, caryophyllene, and estragol. Several groups report that the therapeutic potential of tulsi is mainly for the major bioactive component eugenol (approximately 67%) (Prakash and Gupta, 2005; Pattanayak et al., 2010; Singh et al., 2010). Eugenol is a compound of the phenylpropanoid class, being one of the main components of the essential oil of *Ocimum sanctum*; its chemical structure is shown in Figure 2.

Eugenol is mainly used in food and cosmetics as flavoring agent. It is also known for its excellent antibacterial potential against a wide range of gram-negative (*Escherichia coli*, *Pseudomonas aeruginosa*, *Pseudomonas fluorescens*, *Helicobacter pylori*, *Salmonella typhimurium*) and gram-positive (*Staphylococcus aureus*, *Staphylococcus mutans*) bacteria (Marchese et al., 2017). Eugenol, being hydrophobic and so lipophilic in character, preferentially partitions itself from aqueous phase into bacterial cell membrane. Eugenol affinity to cell membrane causes an increase in membrane permeability and therefore disturbance in ion transport processes; eugenol-mediated inhibition of H^+^ ion transport through cell membrane prevents ATP synthesis and respiratory processes; increased permeability causes cell membrane to lose its integrity and thereby to promote release of intracellular proteins into cell exterior (Gill and Holley, 2004; Kasi et al., 2010). Eugenol, by altering the ion transport through cell membrane, is also capable of generating intracellular ROS, which causes biomacromolecular damages such as DNA degradation, protein oxidation, lipid peroxidation, and ultimately cell death (Marchese et al., 2017). The hydroxyl group of eugenol inhibits the action of protease, histidine carboxylase, and amylase by binding to them in *Enterobacter aerogenes* (Marchese et al., 2017). Eugenol is also reported to reduce significantly in *Staphylococcus aureus* the production of (1) enterotoxins (A and B); (2) toxin 1, the key exotoxin that triggers toxic shock syndrome by inducing TNF-release; and (3) alpha-hemolysin that causes hemolysis of RBC in *Staphylococcus aureus*–infected population (Rafiee et al., 2019). These non-specific actions of eugenol led many laboratories to formulate nano-eugenol, to increase the aqueous solubility and antibacterial potential of eugenol. Different methods of nanonization of eugenol and the antibacterial potencies of the synthesized nano-eugenol have been discussed in the sections that follow.

## Nano-Formulations With Eugenol

Grafting of eugenol molecules on chitosan nanoparticle has been produced through gelation method by Schiff base reaction. According to this method, chitosan-NP is first synthesized by dropwise adding sodium triphosphate to acetic acid solution of chitosan. Eugenol grafting is then performed by adding eugenol into chitosan-NP in presence of methanol under stirring condition (Chen et al., 2009). Such nanoformulation of eugenol is found to have about twofold high antioxidant as well as antibacterial efficacies against *Staphylococcus aureus* and *Escherichia. coli*, compared with raw eugenol in bulk form (Chen et al., 2009). Nanonization of eugenol by entrapping within the synthetic polymer PLGA (poly lactic-co-glycolic acid) has been achieved by solvent evaporation technique, where organic and aqueous phases are emulsified homogeneously through sonication, to remove the organic solvent and finally to produce the polymer-capped NP. Here, organic phase contains PLGA, eugenol, and dichloromethane whereas aqueous phase contains surfactant poly vinyl-alcohol (PVA) as the stabilizing agent and these eugenol-entrapped PLGA-NP has 10 times more inhibitory role than free eugenol on the growth of *Salmonella typhimurium*, *Listeria* monocytogenes, and *Listeria innocua* (Gomes et al., 2011). Solvent evaporation method has also been used to synthesize eugenol-loaded zein (a protein in maize) NP; zein-NP represents a biocompatible carrier for bioactive ingredients. Here, organic phase contains zein and eugenol while aqueous phase contains the surfactant pluronic F 68. Zein nanoparticle-based highly monodispersed eugenol nanoformulation of size 150 nm and zeta potential 30 mV has high encapsulation efficiency (more than 90%) and also exerts a promising bactericidal activity against fish pathogenic bacteria *Aeromonas hydrophila*, *Edwardsiella tarda*, and *Streptococcus iniae* with less toxicity in fishes (Luis et al., 2020). Eugenol-entrapped ethosome nanoparticle (ELG-NP) has been synthesized by ethosome preparation, using eugenol, ethanol, and lipid lecithin as precursors. During preparation of ELG-NP, ethanol, eugenol, and Tween 80 (as stabilizing agent) are first mixed; lecithin is then added dropwise under stirring condition to obtain homogenous ELG-NPs. Such nanoformulation has six times more antibacterial potency than free eugenol against fruit anthracnose (dark lesions on fruits)–causing pathogen *Collectotrichum* sp. (Jin et al., 2019). The entrapment efficiency, particle size, and antibacterial activity of ELG-NPs depend on the percentage of reaction ingredients; 0.5% eugenol, 2% lecithin, and 30% ethanol are the optimum concentrations to produce the most effective ELG-NPs of size 44 nm, with 82% entrapment efficiency and more than 93% antibacterial potency (Rodenak-Kladniew et al., 2019). There is a report on the synthesis of a hybrid eugenol/ofloxacin (quinolone antibiotics used to treat pneumonia, cellulitis, and urinary tract infections)–loaded solid-lipid nanoparticle by emulsification technique. In this method, lipid phase containing lipid, eugenol, and ofloxacin is injected into the aqueous phase of surfactant pluronic F 68 under stirring condition to emulsify lipid and aqueous phases. The emulsified mixture is then suddenly freeze dried to solidify the lipid and to finally obtain powder form of the nanoformulation, which is preserved at 5°C. This hybrid solid-lipid nanoparticle exhibits 6–16-fold better therapeutic potential than free eugenol and ofloxacin against *Staphylococcus aureus* and *Pseudomonas aeruginosa* (Rodenak-Kladniew et al., 2019).

Eugenol has the ability to break bacterial communication, termed as “quorum sensing,” and thus inhibits bacterial biofilm formation. Quorum sensing is a kind of regulation of gene expression in response to alteration of cell population density. The sensing system comprises the sensing molecules, called “auto-inducers,” auto-inducers producing proteins (LuxI), and the sensing receptor (LuxR). Gram-negative bacteria produce acyl-homoserine lactone (AHL) as the auto-inducer molecules, while Gram-positive bacteria use oligo-peptides as the same. When cell population reaches a particular threshold density, they produce auto-inducers, which are transported to the exterior of the cells by passive diffusion (for Gram-negative bacteria) or by ATP-binding cassette-transporter system (for Gram-positive bacteria). When extracellular concentration of auto-inducer molecules reaches a critical value, they bind to their sensing receptors of cells to activate the downstream signaling events to produce virulent factors and thus disease progression (Pena et al., 2019). In case of biofilm infection, eugenol inhibits the synthesis and or competes with auto-inducer molecule to bind with quorum sensing receptor and consequently inhibiting the downstream signaling events. Beside this, eugenol also modulates the conformation of LuxR so that auto-inducer cannot bind properly to the receptor molecule. Inhibition of quorum sensing leads to reduce the production of virulence factors such as pyocyanin, elastase, rhamnolipid, and extracellular polysaccharide in *Pseudomonas aeruginosa* (Rathinam et al., 2017). *Pseudomonas aeruginosa* contains three well-known quorum sensing systems: LasI/LasR, RhlI/RhlR, and PQS (Pseudomonas quinolone signal)/PqsR (Pseudomonas quinolone signal receptor). Nanoemulsion of eugenol and eugenol alone reduce the expression of quorum sensing–related genes in *Pseudomonas aeruginosa* and, therefore, production of virulent factors is also reduced. At a concentration of 0.2 mg/ml, nanoformulation and free eugenol display expression level to be respectively, 52 and 65% for LasI, 45 and 61% for RhlI, and 51 and 65% for RhlA, compared with untreated control *P. aeruginosa* cells. Therefore, nanoemulsion of eugenol exhibits inhibitive effects approximately 13–16% more than free eugenol on quorum sensing–related proteins (Lou et al., 2019). LasA and RhlA encode enzymes such as LasA protease, elastase, and rhamnolipid synthase, which are transcriptionally regulated by quorum sensing and are actively involved in exhibiting virulence in chronic biofilm infection (Adonizio et al., 2008). The oil-in-water nanoemulsion is prepared by mixing pure eugenol, medium-chain triglycerides, surfactant Tween 80, and phosphate buffered saline. Oil phase contains eugenol and medium-chain triglyceride, in which surfactant Tween 80 is injected under sonication to obtain a homogenous mixture and then the mixture is slowly titrated with 88% phosphate buffered saline for 30 min to develop well-dispersed nanoemulsion of eugenol, which shows an inhibitory effect on quorum sensing–associated virulence factors to inhibit biofilm formation by *Pseudomonas aeruginosa* (Lou et al., 2019).

## Turmeric (*Curcuma longa*)

The plant generally grows in warm climate, especially in India and many other parts in Asia (Lakshmi et al., 2011). Actually, the rhizome of the plant is used in spices and medicine. In folk and traditional medicine, turmeric had been used for its versatile therapeutic profile over the centuries in different parts of the world. It is believed from practical experience that turmeric strengthens the human body with overall energy, relieves the body from inherently produced gas, dispels worms, improves digestion, regulates menstruation, dissolves gallstones, and relieves arthritis. From ancient times to the modern era, turmeric is very much popular to treat sprains and swelling. Of the plant-based medicines, turmeric has a multitude of pharmacological properties and is used to combat various microbial diseases like runny nose, cough, sinusitis, and respiratory complications (asthma, bronchial hyperactivity), together with other diseases like liver disorders, anorexia, rheumatism, diabetes, and diabetic wounds, and even cancer also (Luthra et al., 2001). Various multinational companies are using turmeric to prepare face creams because of its significant antibacterial activities (Rafiee et al., 2019).

The excellent pharmacological activities of turmeric have led many laboratories to use it as stabilizing/encapsulating agent for synthesis of metal NPs and to investigate whether the individual antibacterial potency of metal NP (prepared otherwise) and turmeric gets synergistically enhanced for the turmeric-stabilized/encapsulated metallic NPs. Turmeric powder has good potential to reduce metal salts into elemental metal. In hydrothermal method of synthesis of silver-NP, turmeric extract is added into silver nitrate solution at high temperature to obtain highly monodispersed turmeric-stabilized AgNPs. The size of the prepared AgNPs decreases with the increase of reaction time and temperature and their antibacterial activity against *Escherichia coli* O157:H7 and *Listeria monocytogenes* is found to depend on size of the particles (Nayak et al., 2017; Alsammarraie et al., 2018). Such AgNP-impregnated cotton gauze exhibits a strong antibacterial potential against *Staphylococcus aureus*, *Streptococcus pyogenes*, and *Pseudomonas aeruginosa* (Maghimaa and Alharbi, 2020). Copper nanoparticle (CuNP) has also been synthesized using turmeric extract as stabilizing/capping agents. Turmeric reduces copper sulfate to elemental copper, producing highly effective CuNP. The antibacterial efficacy against *Staphylococcus aureus* is determined by disc diffusion on agar plate technique; turmeric-stabilized CuNP exhibits zone of inhibition of 14 mm size, whereas standard antibiotics like ampicillin, methicillin, and penicillin exhibit the size as 12, 10, and 11 mm, respectively (Varghese et al., 2020).

**FIGURE 3:**
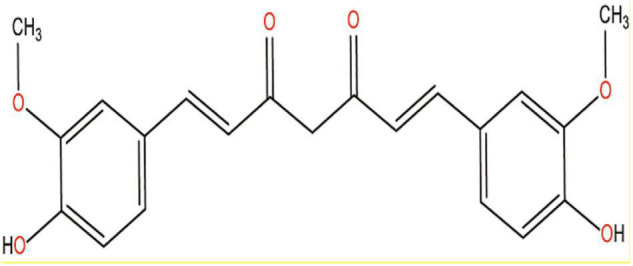
Chemical structure of curcumin.

Of the different parts of the turmeric plant, rhizome has the most pharmacological properties. Rhizome contains a number of bioactive compounds including volatile curcuminoids such as curcumin, demethoxycurcumin, and bisdemethoxy-curcumin and also some volatile oils such as monoterpenoids and sesquiterpenoids (Sharifi-Rad et al., 2020). The therapeutic potentials of turmeric powder are mainly attributed to the major bioactive compound curcumin, which was first isolated in 1,870 (Sharifi-Rad et al., 2020). Curcumin is a naturally occurring yellow-orange-colored, water-insoluble, highly potent polyphenolic compound and exhibits keto-enol tautomerism, but keto form is predominant in neutral and acidic medium (Anand et al., 2007; Yang et al., 2020). The chemical structure of curcumin is shown in Figure 3.

Curcumin is widely used in multiple medicinal formulations over thousands of years because of its multi-medicinal properties such as antimicrobial, antioxidant, anti-inflammatory, anticancer, antirheumatic, cardioprotective, neuroprotective, hepatoprotective, and kidney protective roles; therefore, curcumin is universally known as the “wonder drug of life” (Yeung et al., 2019). To make the hydrophobic curcumin water soluble and to enhance its antibacterial potency, attempts have been made for its nanonization.

## Nano-Formulations With Curcumin

Curcumin nanoparticles of size 2–40 nm have been produced by wet milling method, using curcumin, dichloromethane, and hot water. Here, curcumin in dichloromethane is sprayed into hot boiling water under sonication at room temperature to obtain a clear orange-colored solution, which is then lyophilized to powder of curcumin NP. So far as its antibacterial potency is concerned, a concentration of 400 μg/ml of nanocurcumin exhibits a zone of inhibition of size 12, 14, 16, and 20 mm, whereas the equivalent concentration of free curcumin exhibits the corresponding sizes of 9, 10, 12, and 15 mm against *Escherichia. coli*, *Pseudomonas aeruginosa*, *Staphylococcus aureus*, and *Bacillus subtilis*, respectively (Bhawana et al., 2011; Sun et al., 2014). Curcumin NP, as solid dispersion (CSD), has been synthesized by solvent evaporation technique also. In this method, curcumin and poly (vinyl pyrolodine)-K30 are first dissolved in ethanol and then solidified by lowering the temperature to get CSD. CSD is subsequently loaded in hydrogel by simple addition of CSD into aqueous solution of surfactants P407 and P188 (Adahoun et al., 2017). CSD-loaded hydrogel gives a positive result for the treatment of injured vaginal bacterial infection caused by *Staphylococcus aureus* and *Escherichia coli* and also in the improvement of wound healing (Zhang et al., 2019). Curcumin nanoparticle is also formed by sol–gel method, where tertramethyl orthosilicate is first hydrolyzed by HCl and then the hydrolyzed product is added into chitosan solution of curcumin and finally the whole mixture is lyophilized to get sol–gel-based curcumin nanoparticle (Church et al., 2006). This nanoparticle is found to be highly effective against methicillin-resistant *Staphylococcus aureus* and *Pseudomonas aeruginosa* (Krausz et al., 2015). Combined formulation of AgNPs and curcumin NPs, prepared by mixing AgNPs (synthesized from phytoreduction method by gallic acid; Loo et al., 2014) and curcumin NPs (synthesized from anti-solvent precipitation method by the non-ionic surfactant Pluronic-F127; Lee et al., 2015), exhibits a synergistic action on biofilm formed by drug-resistant gram-positive *Staphylococcus aureus* and gram-negative *Pseudomonas aeruginosa* (Loo et al., 2016).

So far as the mechanism of antibacterial action of curcumin is concerned, it is suggested that the hydrophobic curcumin gets inserted into the membrane in a trans-bilayer fashion, being anchored by hydrogen and phosphate group of lipids and thereby disrupts cell membrane with leakage of intracellular contents (Tyagi et al., 2015). Curcumin suppresses the assembly of cytoskeleton protein FtsZ (filamentous temperature sensitive protein Z) at the junction of two daughter bacterial cells, leading to interruption of bacterial cell division and development of cell filamentation (Kaur et al., 2010). Curcumin is also found to downregulate the expression of RecA and LexA proteins, affecting the LexA–RecA pathway responsible for self-cleavage of DNA during cellular SOS DNA repair process (Li et al., 2018). Furthermore, curcumin downregulates the expression of *srcA* and *srcB* genes involved in sucrose metabolism. Such low level of carbohydrate metabolism results in low secretion of polysaccharides to extracellular surface, causing loss of cellular stickiness and thus cellular ability of adherence to surfaces (Li et al., 2018).

## Aloe Vera

Aloe vera plant has a long history of medicinal use, and it is very much popular for its anti-inflammatory and soothing effects on minor skin cut and burn (Cathcart and Stebbing, 2016). It is believed that the uses of aloe vera were mentioned in Rig Veda, the earliest pre-historic (about 3,000 BC) Indian corpus of natural medicine (Roy, 2011). It was also widely used in Egyptian, Roman, Greek, and other contemporary civilizations for medicinal purposes (Surjushe et al., 2008). Aloe vera has numerous clinical potentials like antibacterial, antifungal, antiviral, anti-inflammatory, and anticancer. It is also applied to treat psoriasis, sunburn or radiation-related dermatitis, esophagitis, allergy, rheumatic fever, rheumatoid arthritis, ulcers, and diabetes. It also lowers blood glucose level and improves the immune system (Zagora-Dziok et al., 2017).

Like other medicinal plant extracts, aloe vera extract (ALE) has also the capability of reducing silver nitrate to produce AgNPs. Antibacterial action of ALE-based AgNPs against *Bacillus subtilis*, *Klebsiella pneumoniae*, and *Salmonella typhi* has been measured, by the disc diffusion method, to be very effective. Such AgNPs also show promising inhibitory actions against *Kocuria varians*, which causes infective endocarditis, arthritis, pneumonia, peritonitis, hepatic abscess, catheter-associated bacteremia, canaliculitis, cholecystitis, dacryocystitis, brain abscess, and meningitis (Velez et al., 2018). ALE also reduces the aqueous solution of CuNO_3_ and develops brick-red-colored ALE-capped copper oxide nanoparticles (ALE-CuONPs) of size about 100 nm, and this nanoformulation exhibits antibacterial activity against fish pathogens *Aeromonas hydrophila*, *Pseudomonas fluorescens*, and *Flavobacterium branchiophilum* (Vijay Kumar et al., 2015). ALE-capped iron nanoparticles (ALE-FeNPs) of size 34 nm can be synthesized by reduction of FeCl_3_ by ALE, and they are very much effective to inhibit the growth of *Proteus mirabilis*, *Salmonella typhi*, and *Shigella flexneri* (Yadav and Kumar, 2016). The reduction of ZnSO_4_ by ALE in the range of pH 5–10 under stirring condition produces ALE-capped zinc oxide nanoparticles (ZnONPs), which are reported to have significant antibacterial activity against extended spectrum of beta lactamase (EsβL)–positive *Escherichia coli*, *Pseudomonas aeruginosa*, and methicillin-resistant *Staphylococcus aureus* (Ali et al., 2016). The common way to exert bacterial toxicity by nanoparticles is generation of reactive oxygen species (ROS) and subsequent occurrence of ROS-mediated phenomena like lipid peroxidation, protein oxidation, and DNA fragmentation with ultimate termination of cellular life (Xia et al., 2008).

**FIGURE 4:**
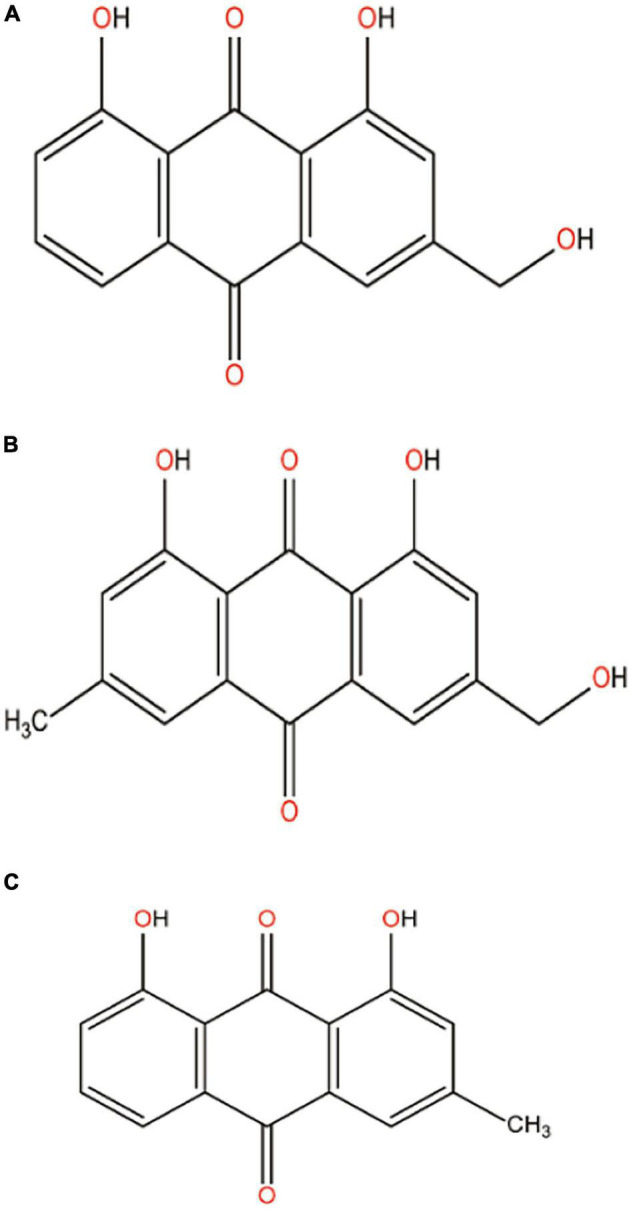
Chemical structure of **(A)** aloe-emodin, **(B)** emodin, and **(C)** chrysophanol.

Aloe vera extract contains about 75 active constituents including vitamins (A, C, E, B_12_, folic acid, and choline), enzymes (alliiase, alkaline phosphatase, amylase, bradykinase, carboxypeptidase, catalase, cellulase, lipase, and peroxidase), minerals (calcium, chromium, copper, magnesium, potassium, sodium, and zinc), sugar, fatty acids, hormones, and anthraquinones. Therapeutic potentials of aloe vera is mainly attributed to the major bioactive compound class anthraquinones (Surjushe et al., 2008) and the main anthraquinones are aloe-emodin (26.29%), emodin (65.30%), and chrysophanol (8.41%) (Kang et al., 2016). These are polycyclic aromatic hydrocarbons, having chemical structures shown in Figure 4. These natural colorants are mainly used in the food industry for food packaging because of their excellent antibacterial activity. Of the three anthraquinone derivatives in aloe vera extract, since the relative concentration of emodin is considerably high compared with the other two derivatives aloe-emodin and chrysophanol, it is therefore expected that the antibacterial efficacy of the aloe vera extract is primarily due to emodin content. Apart from antibacterial activity, the compounds have multiple therapeutic potentials like laxatives, anti-inflammatory, and anticancer, and are also used to treat constipation, arthritis, and multiple sclerosis (Malik and Müller, 2016).

## Nanoformulation With Anthraquinone

Anthraquinone-loaded chitosan-PLA (polylactic acid) nanoparticles (AQ-CS-PLA) are prepared by dropping method, where chitosan-PLA (CS-PLA) nanoparticles are first synthesized by simple mixing of aqueous solutions of chitosan and PLA and anthraquinone is then added into CS-PLA nanoparticles drop-wise (Dhanapal et al., 2014). Anthraquinone-loaded CS-PLA nanoparticles exhibit strong antibacterial activity against *Pseudomonas aeruginosa*, *Klebsiella pneumoniae*, *Proteus vulgaris*, and *Escherichia coli* (Dhanapal et al., 2014). No other report, so far our knowledge goes, has been found to come out on nanonization of any anthraquinone with respect to anti-bacterial action.

## Oregano (*Oregano vulgare*)

It is a small herb 1–3 feet long with olive-green-colored leaves and purple-colored flower. This herb belongs to the mint (Lamiaceae) family, generally found in temperate Himalayas. Both fresh and dry leaves are used in kitchen because of its refreshing flavor and fragrance, which make the recipe more delicious. This herb is also very much common in Greek, Roman, and Mediterranean diet from ancient period. The uses of oregano have been explored worldwide in very recent times after the evaluation of its medicinal properties. In traditional folk medicine, oregano is used to treat microbial infections like colic, cough, headache, and toothaches. It is also used to treat nervousness and irregular menstrual cycle (Leyva-Lopez et al., 2017). Oregano extract contains many phytochemicals, most of which are essential oils such as carvacrol, thymol, γ-terpenene, *p*-cymene, linalool, terpinene-4-ol, β-myrcene, *trans*-sabinene hydrate, and β-caryophyllene (Leyva-Lopez et al., 2017). Essential oils of oregano are extremely popular for fragrance and for their excellent antimicrobial properties; they also have various other pharmacological activities such as highly antioxidant, anti-inflammatory, cardio-protective, and metabolism aids (Leyva-Lopez et al., 2017).

Oregano leaf extract (OLE) has been used to produce silver-NP by hot hydrothermal method, where silver nitrate is reduced to metallic silver by the antioxidant property of OLE, and these OLE-stabilized silver nanoparticles (OLE-AgNPs) show high antibacterial activity against *Pseudomonas aeruginosa* and *Staphylococcus aureus* (Meretoudi et al., 2021). Gold nanoparticles (AuNPs) can also be prepared with the help of polyethylene glycol (PEG) and oregano extract. During nanoformulation, HAuCl_4_ is used as precursors and OLE as indicator whereas PEG is used as both reducing and capping agents. In presence of Au^3+^, oregano extract gives an indicative ruby red color to the nanoformulation OLE-AuNPs. Oregano synergizes the antibacterial potential of AuNPs, and OLE-AuNPs act as effective antibacterial against gram-positive bacteria *Staphylococcus aureus* ATCC 6538P, *Listeria monocytogenes* ATCC13932 as well as gram-negative bacteria *Salmonella enteritidis* ATCC 13076 and *Escherichia coli* ATCC 25922, when determined by disc diffusion method (Benedec et al., 2018). Nanonization of oregano oil has been made by the “oil in water” nanoemulsion method by simply mixing oregano oil and the surfactant Tween 80 in water in 2:1 ratio, followed by sonication for 10 min, and this nanoformulation is found to control foodborne bacterial pathogens *Listeria monocytogenes* ATCC 19115, *Salmonella typhimurium* ATCC 19585, and *Escherichia coli* O157:H7 ATCC 700927 on lettuce leaves (Bhargava et al., 2015). Nanoemulsion of oregano oil has also been synthesized by phase inversion method, where different amounts of oregano oil and surfactant in water are mixed and then subjected to heating/cooling cycle twice to encapsulate oregano oil within surfactant. The droplet size of nanoemulsion depends on the amount of oil. Such nanoemulsion of oregano oil has significant antibacterial activity on *Staphylococcus aureus* and *Escherichia coli* and may be used as food preservative (Moraes-Lovison et al., 2017). The nanoemulsion-incorporated hydroxypropyl methyl cellulose–based active nanocomposite exhibits strong antioxidant and antibacterial properties against *Salmonella* sp. (Lee et al., 2019). Oregano extract inhibits bacterial attachment, motility, and production of a virulent factor “shiga toxin” by downregulating the expression of the corresponding genes *ler*, *fliC*, and *stx2B* in *Escherichia coli* EHEC O157:H7 (Barbosa et al., 2020). Oregano oils weaken the cellular membrane leading to leakage of small molecules such as Na^+^, K^+^, Ca^2+^, H^+^, and Cl^–^ and thereby causing inhibition of membrane coupled energy production and finally cell death (Mith et al., 2015).

**FIGURE 5:**
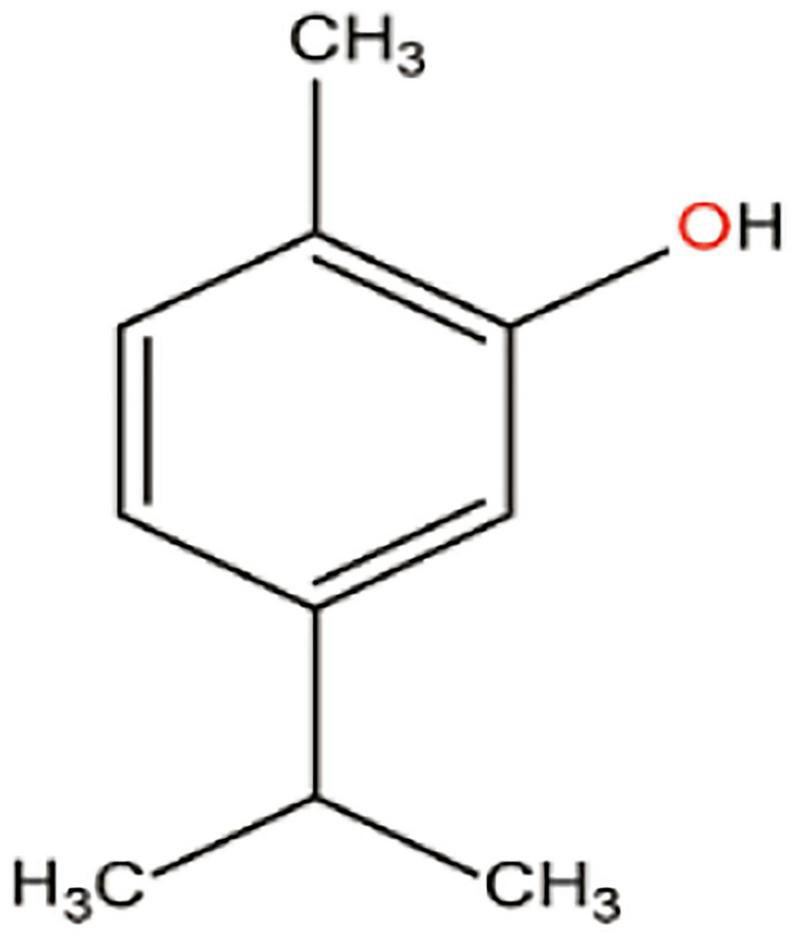
Chemical structure of carvacrol.

Oregano leaf extract contains principally essential oils. Oregano essential oils are carvacrol, β-fenchyl alcohol, thymol, and γ-terpenine. Therapeutic potentials of oregano are mainly attributed to its major component carvacrol, which belongs to phenolic monoterpenoid compound (Leyva-Lopez et al., 2017; Figure 5), found mainly in oregano, thyme, and peppermint plants (Sharifi-Rad et al., 2018). This compound possesses numerous biological activities including antibacterial, antifungal, antiviral, anticancer, antigenotoxic, antispasmodic, anti-inflammatory, antiparasitic, anti-elastase, hepatoprotective, AChe inhibitory, and food additives (Baser, 2008). Carvacrol is very much popular in the food industry due to its flavor and significant antibacterial activities (Ozkan et al., 2017). The hydrophobic nature of carvacrol led to the synthesis of its nano-form to develop its aqueous solubility and therefore bioavailability (Figure 5).

## Nanoformulation With Carvacrol

Carvacrol-loaded PLGA nanoparticles have been prepared by solvent displacement method. In this method, PLGA, surfactant epikuron, and carvacrol are first dissolved in acetone; the mixture is then added into aqueous solution of another surfactant pluronic F65 and the whole mixture is finally concentrated by applying pressure to obtain carvacrol-loaded nanoparticles. PLGA acts as a capping agent while both surfactants are used as stabilizing agents to prepare carvacrol-loaded nanoparticles, which are found to possess strong antibiofilm activities formed by *Staphylococcus epidermidis* ATCC 35984 (Iannitelli et al., 2011). Carvacrol-loaded ovalbumin gel nanoparticles are developed by mixing egg white with carvacrol, heating the mixture for 30 min at 90°C, followed by immediate cooling in an ice bath; the prepared gel is finally mixed by stirring to achieve fabricated and homogenized ovalbumin–carvacrol gel nanoparticles. This nanoformulation displays a promising antibacterial activity against *Salmonella* sp. and *Bacillus cereus* (Rao et al., 2020). Synthesis of carvacrol-loaded solid-lipid nanodispersion has been made by microemulsion template method, where lipid phase contains propylene glycol monopalmitate, glycerol monosterate, and carvacrol whereas aqueous phase contains Tween 80; the lipid phase is added dropwise into aqueous phase under continuous stirring to form a transparent microemulsion. This solid-lipid nanodispersion is very much effective to inhibit the growth of gram-negative *Escherichia coli* O157:H7 and gram-positive *Staphylococcus aureus* (He et al., 2019). Carvacrol-loaded polycaprolactone (PCL) nanoparticles are prepared by nanoprecipitation method. Polymer PCL is dissolved in acetone, carvacrol is then added into PCL solution with mild heating, and the mixture is then added into aqueous phase of surfactant polaxamer followed by homogenization and centrifugation. This nanoformulation is found to be effective against *Pseudomonas aeruginosa* and *Staphylococcus aureus* (Mir et al., 2020).

The promising antibacterial activity of carvacrol is attributed to the permeabilization and depolarization of cell membrane (Xu et al., 2008). Carvacrol exhibits rapid bactericidal activity against pathogens like *Escherichia coli* and *Streptococcus pyogenes* through cell membrane damage and consequent inhibition of some cellular processes like syntheses of DNA and lipid, and leakage of cytoplasmic content such as lactate dehydrogenase enzymes and nucleic acids (Wijesundara et al., 2021). It is also reported that carvacrol (1) penetrates the bacterial cell membrane easily in *Listeria monocytogenes*, (2) changes the composition of fatty acids that affects membrane fluidity and permeability, (3) causes a decrease in membrane polarity and inhibition of cellular respiratory activity (Churklam et al., 2020), (4) reduces the expression of virulent enterotoxin in *Staphylococcus aureus* by controlling the complex regulatory network (Zhang et al., 2020), and (5) decreases motility in *Salmonella typhimurium* with loss of functionality of flagellum (Zhang et al., 2020).

## Eucalyptus (*Eucalyptus globulus*)

The term eucalyptus was first given and described by French botanist L’Heritier. It is a woody, perennial, and mostly evergreen plant and grows well in deep, fertile, well-drained loamy soil with adequate moisture (Acharya and Acharya, 2019). Eucalyptus is widely used in traditional medicine for its versatile and excellent pharmacological properties. Because of its anti-septic and anti-spasmodic properties, it is mainly used to treat respiratory complications such as bronchitis, asthma, lower respiratory tract infections, and chronic obstructive pulmonary diseases (Horváth and Ács, 2015). It also increases blood flow and skin temperature (Hayat et al., 2015). Eucalyptus extract also exhibits anti-inflammatory, antibacterial, anticancer, and astringent activities (Sulaiman et al., 2013). For its excellent therapeutic activities, leaf and bark extracts of eucalyptus plant have been nanonized by different methods.

It is reported that eucalyptus leaf extract (ELE) produces metallic AgNPs on shaking with silver nitrate solution in a gyratory shaker incubator at 150 rpm and at 28°C in the dark for 16 h. The prepared AgNPs inhibit the growth of multidrug-resistant *Acinetobacter baumannii* isolated from a pneumonia patient (Wintachai et al., 2019). These AgNPs have effective antibacterial activity against pathogenic bacteria *Pseudomonas aeruginosa*, *Escherichia coli*, *Staphylococcus aureus*, and *Bacillus subtilis* also (Sulaiman et al., 2013). ELE-stabilized AgNPs have also been developed by microwave-assisted technique. In this process, ELE and AgNO_3_ are mixed in a conical flask and then subjected to microwave treatment in a domestic microwave oven operating at a power of 8,000 W and frequency 2,450 MHz for a short pulse of 30 s. Such AgNPs have antibacterial and antibiofilm potentials against *Escherichia coli* and *Staphylococcus aureus* (MRSA and MSSA) (Ali et al., 2019). ELE has the potential to reduce Cu (NO_3_)_2_ also, producing CuO-NPs of size about 27.2 nm, and the particles show excellent inhibitory effect on bacterial biofilm formation (Ali et al., 2015). Not only biofilm cells, ELE-stabilized CuONPs are also more effective than commercially available bulk CuO molecules to kill even the planktonic cells of β-lactamase–producing *Escherichia coli* 336, *Pseudomonas aeruginosa* 621, and methicillin-resistant *Staphylococcus aureus* 1 (Shanan et al., 2018). By the method of phytoreduction, ELE also produces nickel oxide nanoparticles (NiO-NPs) from the precursor nickel hexahydrate (NiNO_3_⋅6H_2_O). These NiO-NPs exhibit antibacterial and antibiofilm potential against EsβL-producing *Escherichia coli*, *Pseudomonas aeruginosa*, and methicillin-resistant and sensitive *Staphylococcus aureus* (Saleem et al., 2017).

**FIGURE 6:**
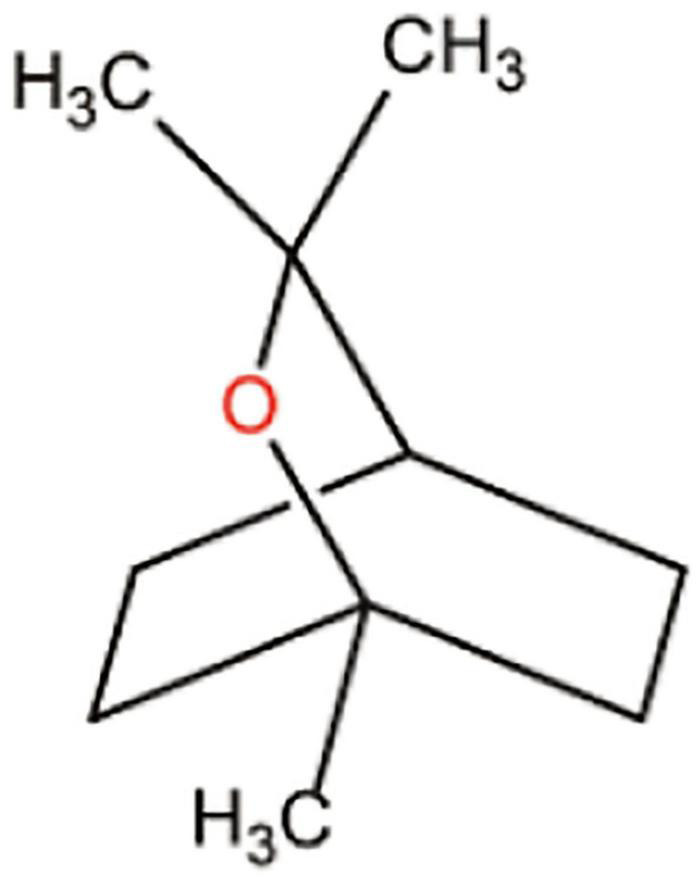
Chemical structure of eucalyptol.

ELE comprises various compounds, of which essential oils are the major part. All the essential oils are collectively termed as eucalyptus oil, which includes eucalyptol (1,8-cineole), *p*-cymene, α-pinene, β-myrcene, and γ-terpinene. Eucalyptol is the major oil component in eucalyptus oil, with the chemical structure shown in Figure 6. The therapeutic potential of ELE is mainly attributed to the bioactive components—eucalyptus oils (Almas et al., 2021). Due to its excellent pharmacological profile, nanoformulation of eucalyptus oil has been made to increase both its bioavailability and pharmacological profile.

## Nanoformulation With Eucalyptus Oil

Nanoemulsion of eucalyptus oil (cineole: 60%) is prepared by sonicating a mixture of eucalyptus oil, Tween 80, and water. The amplitude and sonication time determine the particle size of nanoemulsion. This nanoformulation significantly reduces the population of clinical pathogen *Staphylococcus aureus* and has wound healing activity in Wistar rats (Sugumar et al., 2014). Eucalyptus oil–impregnated chitosan nanoparticle has been found to have more antibacterial potency, compared with free oil, against clinical pathogen *Staphylococcus aureus* (Sugumar et al., 2015). Eucalyptus oil nanoemulsion, synthesized by spontaneous emulsification, which involves simply mixing water into heated organic phase containing eucalyptus oil and surfactant Tween 20 under stirring condition at 400 rpm, is also effective to stop the growth of gram-negative pathogen *Listeria monocytogenes* MTCC 1143 (Chandrasekaran, 2015) and different foodborne pathogens such as *Escherichia coli*, *Staphylococcus aureus*, and *Bacillus cereus* (Pathania et al., 2018). Well-dispersed nanoemulsion of eucalyptus oil has also been prepared by using double surfactants, where the oil phase containing oil and one surfactant sorbitan monooleate is injected into the aqueous phase containing the other surfactant Tween 80, under stirring condition with controlled temperature in an ice bath. This nanoemulsion is very much efficient to stop the growth of *Pseudomonas aeruginosa* (Quatrin et al., 2017).

The exact antibacterial mechanism of eucalyptus oil is not yet fully understood. Exposure of oil increase bacterial cell surface hydrophobicity. As essential oils are hydrophobic in nature, the increased hydrophobicity in cell surface easily allows oil to penetrate the membrane. The oils destabilize the membrane phospholipid bilayer and thus affects various cellular processes, finally causing cell death (Lopez-Romero et al., 2015).

## Conclusion

**FIGURE 7:**
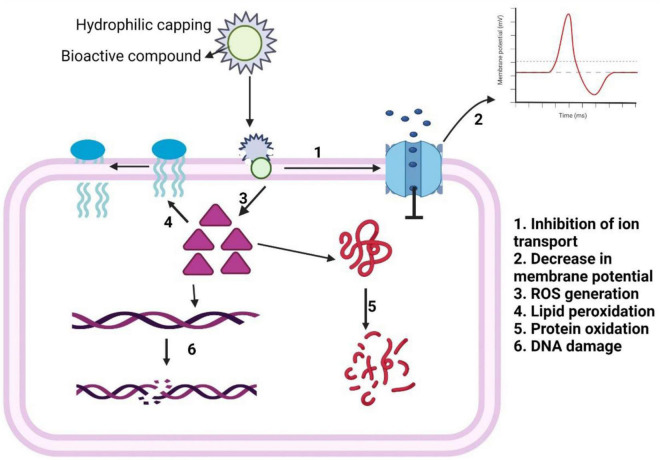
Schematic representation of antibacterial mechanism of action of bioactive compound-loaded nanoparticles.

For thousands of years, natural products have been used to treat various bacterial infections all over the world. Historically, the major contribution in pharmacotherapy and medicine arrived from different medicinal plant products and their structural analogs. However, natural plant products present some technical and biological challenges for being developed as drugs. The technical challenges are barriers to screen the active ingredients of plant extracts through isolation, characterization, and optimization, whereas the biological challenges are their less bioavailability for low aqueous solubility and lack of scientific research outputs on the mechanism of their specific biological actions. For these challenges, pharmaceutical industries decline their interest to pursue natural products as drugs; instead, their R&D emphasis rallied toward chemically synthesized counterparts. Some recent technological and scientific developments on improved analytical tools like GC-MS, (LC-MS)-MS, and NMR spectroscopy fade out the technical challenges, whereas the use of various strategies of nanotechnology makes the plants’ components more bioavailable and more potentially bioactive. Therefore, modern scientific and technological developments vitalize the bioactive components of medicinal plants to evolve out as a new platform of drug development and drug delivery. The combination of natural product and nanotechnology will gradually evolve out nano-naturopathic antibacterial drugs, which have advantageous features such as multiple mechanisms of action, lower possibility of interaction with a particular biomolecule (as in case of antibiotic) and therefore less tendency to induce bacterial resistance, biocompatibility and no cytotoxicity at scheduled dose, less side effects, and better therapeutic potentials due to enhanced surface area to volume ratio of the nanonized plant products, over the bulk form of the same natural products. The gross molecular mechanism of antibacterial action of the nanonized bioactive compounds of medicinal plants, as summarized in Figure 7, can be suggested as follows: the medicinal phytochemicals being lipophilic in nature bind to bacterial cell membrane, causing loss of membrane integrity with consequent decrease of membrane potential, ion transport, and energy production, which in result induces and enhances cellular ROS level that lead to subsequent lipid peroxidation, protein oxidation, DNA damage, and finally cell death. Fundamental understanding on the mechanism of antibacterial action in more molecular detail, through studies on up-/downregulation of bacterial genes, proteins, and metabolites, is yet to be attained and toward which future research should be directed. Table 1 contains the overall extract of information, transpired in this review, about the major phytochemicals of the five different medicinal plants, their nanonization by different established methods, and their target bacteria.

**TABLE 1:** Nanonization of the major component of five different medicinal plants by different established methods of nanonization and their antibacterial potential on different bacteria.

Major component of plant	Method of nanonization	Nanoformulation	Antibacterial action on	References
Eugenol	Ionic gelation and Schiff base reaction Solvent evaporation technique Ethosome Solid-lipid NPs	Eugenol-grafted chitosan NPs Eugenol-loaded PLGA NPs Eugenol-loaded Zein NPs Eugenol-entrapped ethosome NPs Eugenol/ofloxacin Solid-lipid NPs	*S. typhimurium*, *L. monocytogenes*, *L. innocua*, *A. hydrophila*, *E. tarda*, *S. iniae*, *P. aeruginosa*	Chen et al., 2009; Gomes et al., 2011; Rathinam et al., 2017; Jin et al., 2019; Lou et al., 2019; Rodenak-Kladniew et al., 2019; Luis et al., 2020
Curcumin	Wet milling Solvent dispersion Phytoreduction and anti-solvent precipitation Oil-in-water emulsion Sol–gel method	Curcumin NPs CSD AgNPs and curcumin NPs Curcumin microemulsion Curcumin NPs	*E. coli*, *P. aeruginosa* *S. aureus*, *B. subtilis*, *S. epidermidis*, *S. aureus*, *B. subtilis*, *P. aeruginosa*	Church et al., 2006; Kaur et al., 2010; Bhawana et al., 2011; Loo et al., 2014; Sun et al., 2014; Krausz et al., 2015; Lee et al., 2015; Yun and Lee, 2016; Adahoun et al., 2017; Li et al., 2018; Zhang et al., 2019
Anthraquinone	Dropping method	AQ-CS-PLA	*P. aeruginosa*, *K. pneumoniae*, *P. vulgaris*, *E. coli*	Dhanapal et al., 2014
Carvacrol	Solvent displacement Simple mixing Microemulsion	Carvacrol-loaded PLGA NPs Fabricated ovalbumin/carvacrol NPs Carvacrol-loaded solid-lipid nanodispersion	*S. epidermidis*, *S.* sp. *B. cereus* *E. coli O157:H7* *P. aeruginosa* *S. aureus*	Iannitelli et al., 2011; He et al., 2019; Mir et al., 2020; Rao et al., 2020
Eucalyptus oil	Sonication cavitation Impregnated nanoemulsion Nanoemulsion	Eucalyptus oil nanoemulsion Eugenol-impregnated chitosan nanoemulsion Eucalyptus oil nanoemulsion	*L. monocytogenes* *S. aureus* *E. coli* *B. cereus*	Sugumar et al., 2014; Chandrasekaran, 2015; Lopez-Romero et al., 2015; Quatrin et al., 2017

**FIGURE 8:**
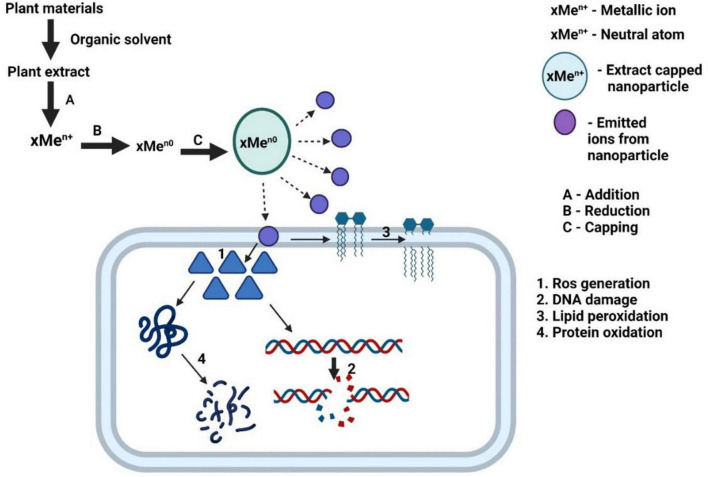
Schematic representation of antibacterial mechanism of action of plant extract stabilized metallic nanoparticle.

In addition to the nanoformulation of the major plant components, use of the plants’ crude extract as reducing/stabilizing/capping agent (s) for preparing different metallic and metal oxide NPs and the antibacterial efficacy of these NPs have also been described in this review. The gross mechanism of antibacterial action of these green synthesized metallic NPs, as summarized in Figure 8, can be interpreted as follows: the plant whole extracts containing different phytochemicals, being a good antioxidant, tend to reduce metal salts to zero-valent metallic NPs, which may further be stabilized and/or capped by the phytochemicals; the NPs, when added in bacterial culture medium, are oxidized and emit metal ions, which cause an increase in cellular ROS level leading to cellular lipid peroxidation, protein oxidation, DNA damage, and ultimately cell death. Table 2 shows a consolidated view of this review coverage on synthesis of different metal and metal oxide NPs, using the five different medicinal plant extracts through different established methods of nanonization and the target bacteria of the NPs.

**TABLE 2:** Green synthesis of different metal and metal oxide NPs, using five different medicinal plant extracts and different established methods of nanonization, having antibacterial potential on different bacteria.

Name of the plant	Method of nanonization	Precursor metallic solution	Nanoformulation	Antibacterial action on	References
Tulsi	Phytoreduction	AgNO_3_	AgNPs	*E. coli*, *S. aureus*, *K. pneumoniae*, *B. linens*, *P. acnes*, *B. cereus*, *S. epidermidis*	Ramteke et al., 2013; Durán et al., 2016; Ramkumar et al., 2017; Khorrami et al., 2018; Jacob et al., 2019; Yin et al., 2020
Turmeric	Phytoreduction	AgNO_3_ CuSO_4_	AgNPs CuNPs	*E. coli* O157:H7, *L. monocytogenes*, *B. subtilis*, *P. aeruginosa*, *S. aureus*	Nayak et al., 2017; Alsammarraie et al., 2018; Maghimaa and Alharbi, 2020; Varghese et al., 2020
Aloe vera	Phytoreduction	AgNO_3_ CuSO_4_ FeCl_3_ ZnSO_4_	AgNPs CuONPs FeNPs ZnONPs	*E. coli*, *P. aeruginosa*, *B. subtilis*, *K. pneumoniae*, *S. typhi*, *K. varians*, *A. hydrophila*, *P. fluorescens*, *F. branchiophilum*, *P. mirabilis*, *S. flexneri*	Xia et al., 2008; Vijay Kumar et al., 2015; Ali et al., 2016; Yadav and Kumar, 2016; Velez et al., 2018
Oregano	Hot hydrothermal method PEG stabilization	AgNO_3_ HAuCl_4_	AgNPs AuNPs	*P. aeruginosa* *S. aureus* *S. enteritidis*	Bhargava et al., 2015; Mith et al., 2015; Moraes-Lovison et al., 2017; Benedec et al., 2018; Lee et al., 2019; Meretoudi et al., 2021
Eucalyptus	Phytoreduction	AgNO_3_ CuSO_4_ NiNO_3_⋅6H_2_O	AgNPs CuONPs NiONPs	*P. aeruginosa*, *E. coli*, *S. aureus*, *B. subtilis*	Sulaiman et al., 2013; Saleem et al., 2017; Ali et al., 2019; Wintachai et al., 2019

So far as our knowledge goes, there is hardly any report to this date on nanonization of whole extract of any medicinal plant organ through encapsulation or entrapment within any capping agent or nanocarrier. In this regard, our venture of nanonization of *Ocimum sanctum* leaf extract, using gelatin as the capping agent, is highly inspiring so far as the role of the nanoformulation against renal lithiasis and bacteriofilm disorders is concerned (unpublished results).

## Author Contributions

SG: manuscript preparation, information collection, and picture preparation. SN: information collection. TB: revision and final manuscript preparation. All authors contributed to the article and approved the submitted version.

## Conflict of Interest

The authors declare that the research was conducted in the absence of any commercial or financial relationships that could be construed as a potential conflict of interest.

## Publisher’s Note

All claims expressed in this article are solely those of the authors and do not necessarily represent those of their affiliated organizations, or those of the publisher, the editors and the reviewers. Any product that may be evaluated in this article, or claim that may be made by its manufacturer, is not guaranteed or endorsed by the publisher.
